# A personal tribute to Sanjiv Sam Gambhir MD PHD

**DOI:** 10.7150/ntno.67358

**Published:** 2022-01-01

**Authors:** Natesh Parashurama

**Affiliations:** 1 Chemical and Biological Engineering, University at Buffalo, State University of New York, Buffalo, New York, USA; 2 Clinical and Translation Research Center (CTRC), University at Buffalo (State University of New York), 875 Ellicott St., Buffalo, NY 14203, USA; 3 Department of Biomedical Engineering, University at Buffalo (State University of New York), Furnas Hall, Buffalo, NY 14260, USA; 4 Center for Cell, Gene and Tissue Engineering, University at Buffalo, State University of New York, Furnas Hall Buffalo NY 14260, USA

All of the former mentees and trainees are heart-broken at the loss of our mentor Sanjiv Sam Gambhir in July 2020. We are all still mourning and will be for some time. Together with the Stanford Radiology Department and School of Medicine, we recently organized and participated in the first annual Sanjiv Sam Gambhir Symposium in July of 2021, with hopefully many more to follow. I am writing this tribute because I feel I can provide a unique glimpse of Sam as a postdoc with Sam, because I was assimilating molecular imaging for the first time, and because I am a physician scientist with a background in physical sciences/ engineering as he was. I am honored to provide unique insight to this academic giant in this tribute.

Prior to meeting Sam, most physician scientists that I had met previously had a disease focus and were focused basic scientists; however, they would never think about how to translate their ideas in patients, nor would they actually succeed at it. Thus, they would never go past *in vitro* or *in vivo* rodent studies. Many scientists or clinicians were often not willing to put the effort in to write IND applications, work with FDA, perform toxicity studies, and help lead clinical trials. I was wrong, as these efforts were a central component of Sam's research. Sam applied his interdisciplinary background to work on the whole body, not a specific tissue or molecule or disease mechanism. He boldly created translational research ideas and constantly worked out how they would look in a patient. He believed in science without borders, in new machines and technology replacing old ones, and a vision of medicine in which disease was detected at its earliest stages. His knowledge of translational medicine was well ahead of its time, and it could not be learned in any textbook or course. Consistent with this theme, I had the fortune of organizing and conducting a high risk, large animal, collaborative (Drs. Michael McConnell and Phil Yang, Cardiology), cardiac cell imaging and therapy study in the lab, “the pig study,” for which the president of the university had to sign off on the animal protocol, and for which I had to take over our lab's culture facilities to grow 2 billion cells. In doing so, I felt his passion for clinical translation, and for overcoming any obstacle in his path.

A common theme that ran through nearly all of his research was the concept of interrogating the human (or mouse) body for information regarding its health and disease state, which was constantly being communicated by the body. Perhaps because of this concept he had a unique approach compared to many scientists, as he didn't get caught in the trap of single school of thought or a single imaging modality, and always realized the larger picture. He didn't worry about whether angiogenesis drugs were effective, but instead co-developed molecular probes to monitor these therapies and detect cancers. He wasn't concerned whether a particular cell/gene therapy would be better than another, instead he focused is research on co-conducting the first reporter gene imaging trial in patients, and the clinically translating reporter gene imaging to patients. His lab even developed techniques to image RNA *in vivo*. Similarly, whether a particular, chemical-, nano-, or protein-engineered probe was selected as the final probe was not his concern. Instead, he was much more interested in whether PET imaging of the probe demonstrated favorable vs. unfavorable pharmacokinetics, which would either advance or preclude their use, respectively.

Similarly, whether it was a Raman or Photoacoustic endoscope or an optical biosensor, he was passionate about technology, but didn't get enamored with a particular imaging approach. This approach led him to pioneer simple concepts in multimodality imaging that are still not yet used, by combining the strengths of different imaging modalities, rather than trying to create the perfect imaging technology. Using his deep knowledge of the limitations and strengths of each approach, he leveraged this creatively to manifest completely new methods or strategies. As part of my “pig study,” I was fortunate enough to experience his passion for multimodality imaging, as we combined fluorescence, bioluminescence, MRI, and PET in a single study for cell imaging, considering the strengths and weaknesses of each technique. I also participated in a collaboration with Electrical Engineering (Dr. James Harris) where we co-developed miniature sensors for *in vivo* detection, a project that exhibited his love of the development of new technology.

Unlike many other labs or research groups, many of the projects in our lab were extremely difficult and times nearly impossible. The projects often had nothing in common with each other, in terms of research questions, methods, techniques, or experimental systems. As soon as I thought he was interested in breast cancer, he was also focusing on prostate or gut cancer, gene therapy to the liver, or immuno-oncology. We must have had 150 projects in the lab, and this list was probably much larger over all the years, with most of them having collaborators. He was not attached to any single project, as he thought all of them were valuable. Nevertheless, he was able to guide the projects with his understanding of the fundamental physics of each of these devices, the fundamental biology or chemistry of probes, and a focused strategy that he developed. Our lab was extremely interdisciplinary, as we had molecular and cell biologists, radiochemists, chemical biologists, nano-synthetic chemists, physicists, imaging scientists, protein engineers, bioengineers, instrumentation engineers, material scientists, and doctors from all over the world in the lab. We performed a wide ranging and long list of techniques that matched the background of these scientists. In the midst of managing all these projects, as well as all of his administrative duties and clinical duties, Sam found the time to be an expert in cancer, oncology, and immuno-oncology. Within the same sentence, he would quickly address details within biology, engineering, or medicine. He realized that innovation lies between these normally disparate disciplines. I experienced this directly, when he pushed me to create new methods to image single breast cancer stem cells *in vivo*, together with our collaborator Michael Clarke at the Stem cell institute, which also demonstrated to me how he enjoyed stretching a technology to its limits.

He exhibited many other valuable qualities worth mentioning. He was very, very, collaborative, to an extreme. As was mentioned above, I started projects with medicine (Cardiology/ Cardiac Surgery), Biology (The Stem Cell Institute), and Electrical Engineering. These three disparate departments represented a truly small sample size of the academic and industrial collaborators he had across campus and the world. We are still trying to figure out his impact worldwide. When I had to present at the Molecular imaging conference in Montreal, he wanted to review my slides, as he did with all the new postdocs. However, every single person at the conference wanted to meet with him for their own particular reasons, often because they wanted to collaborate with him. I was surprised when, instead of meeting and chatting with his colleagues at the conference, he was still focused on the quality of my presentation; in an effort to find a quiet area, he led me to walk well past the main entrance and into a maze of hallways to find a distant, silent, couch in the corner of the conference hall to review my slides. The fact that he did not mind doing this was truly shocking to me. It revealed to me that his focus was always on research. At the same conference, I was on the flight back with him, and he was working on a grant in the gate and on the plane, where I sat next to him. Even though he quietly worked the entire time while everyone rested or watched a movie, he told me when we arrived at SFO that “I got some work done, but I couldn't focus.” Actually, all he did was focus, as I was reading a book on the plane, and all he did the entire flight was work on the word document. Not only was he collaborative, but he had a great reputation enough to warrant his collaborations which many of us carried out, and because he was disciplined, and he had high character, and he liked managing problems and situations that arose, rather than running from them like most people would.

Like all great leaders, he brought together disparate “tribes” of scientists under the common bond of molecular imaging, whether it was therapeutic monitoring, or early detection, material scientists or molecular biologists. He invented terminology and built a language around molecular imaging, early detection and translational medicine that I never have seen or will see in any textbook. He always had clear vision for the future of medicine. Not only did he possess numerous leadership skills, he was also a thought leader who influenced pharmaceutical and imaging companies, academic centers, and the NIH. To reach this level of achievement took a high level of confidence, which he naturally exhibited, and ultimately he realized the greater battle was that we were all attempting to battle disease by detecting it early, rather than just trying to cure it. He had a sense of what hard work was and he expected that from all. He was also a great educator, and used to spend time after lab meeting for the new postdocs and students going over imaging fundamentals. He was a great orator who never stumbled on his words, and presented complex ideas in a very digestible way. He expected out of a lot of us. When we joined the lab, we quickly realized that we were not all in a regular lab, but rather we were in a formal training program in molecular imaging. I knew this because the first day we were handed a huge 1000 page binder (eventually turned into his textbooks) that we were expected to read cover to cover. He also shared the entrepreneurial spirit that pervades Stanford university, and very frequently worked with the intellectual property office, and we had the fortune of being on a patent application together.

Sam's lab was very productive, publishing a manuscript a week, because he uniquely figured out how to manage research, something that is very hard to do, and how to get maximal productivity out of his lab. I remember he told me that other scientists let their lab just run itself, but he liked running the lab. He was organized enough to easily run and manage the projects in the lab, and to handle thousands of emails every day. This is what enabled him to hold so many leadership positions simultaneously. There were times I would be in his office talking about an experiment to do, and then a phone call from the clinic would interrupt our conversation and he would be addressing critical issues that a nurse had with a patient in the imaging clinic. Then 30 seconds later we would be back talking about an experimental detail. There was no limit to his ability to multi-tasking capacity.

I was fortunate to work with him at this height of his career, and I will never forget it, between 2008-2012, and was in touch with him afterwards until the end. I remember, I was struggling to write our first manuscript, and I told him once that I would give him my car keys if I didn't get the manuscript done on time. I remember I sent it to him three hours late, but it was because of software-related problems. Thankfully, I kept my keys. I can only hope now Sam is comfortable, and has the keys now to a much grander car.

